# Effects of Substituting the Two-Spotted Cricket (*Gryllus bimaculatus*) Meal for Fish Meal on Growth Performances and Digestibility of Striped Snakehead (*Channa striata*) Juveniles

**DOI:** 10.3390/life13020594

**Published:** 2023-02-20

**Authors:** Noratat Prachom, Bundit Yuangsoi, Jarongsak Pumnuan, Mohamed Ashour, Simon J. Davies, Ehab El-Haroun

**Affiliations:** 1 Department of Aquaculture, Kasetsart University, Bangkok 10900, Thailand; 2 Faculty of Agricultural Technology, King Mongkut’s Institute of Technology Ladkrabang, Bangkok 10520, Thailand; 3 Faculty of Agriculture, Khonkaen University, Khon Kaen 40002, Thailand; 4 National Institute of Oceanography and Fisheries (NIOF), Cairo 11516, Egypt; 5 Aquaculture Nutrition Research Unit (ANRU), Carna Marine Station, College of Science & Engineering, Ryan Institute, The University of Galway, H91 TK33 Galway, Ireland; 6 Fish Nutrition Research Laboratory, Faculty of Agriculture, Cairo University, Cairo 12613, Egypt

**Keywords:** fish meal, alternative protein, insect meal, cricket meal, *Channa striata*

## Abstract

This study aimed to investigate the potential of using field two-spotted cricket *Gryllus bimaculatus* as the main protein source in fish feed for striped snakehead (*Channa striata*) juveniles. A 10-week feeding effect on growth performance, feed utilization, digestibility of major nutrients, including amino acids, and physiological outputs of nitrogen and phosphorus were determined. A total of 225 *C. striata* juvenile fish (Initial weight, 15.0 ± 0.1 g) were randomly distributed into three dietary groups in triplicate (25 fish per rectangular aquarium within a semi-recirculating system). Each group was hand-fed one of the experimental diets containing the graded level of a cricket meal (CM) replacing 0%, 50%, and 100% (CM_0%_, CM_50%_, and CM_100%_, respectively) of fish meal (FM) protein component. The results showed that growth performance and protein retention tended to increase with increasing dietary CM levels, whereas the waste outputs of nitrogen (N) and phosphorus (P) decreased. Apparent net protein utilization (ANPU) and P retention values increased with increasing levels of cricket meal inclusion level in the diet. There was a significant reduction in both N and P solid waste and dissolved waste output for snakehead with increased CM inclusion. There were significant effects of CM level on fish whole-body composition in terms of elevated protein and fat content. In conclusion, the CM is a viable alternative protein source for aquaculture feeds and can be included up to 100% as a replacement for FM without compromising the growth performance of striped snakehead *Channa striata* juveniles. This may also have a more favorable impact, with the potential to reduce N and P loading to the environment.

## 1. Introduction

The striped snakehead (*Channa striata*) is a commercially valued fish species in Asia, especially Thailand, the Philippines, Cambodia, and Vietnam [1]. The farming of snakeheads was commercially started in 1990, but breeding has been successfully established in the last decade. Snakehead production was mainly collected from inland fisheries at around 92.8%, and the remaining was from intensive aquaculture [2]. The snakehead is an opportunistic carnivorous fish eating frogs, snakes, insects, earthworms, and tadpoles, and it was considered to be a pest, prompting eradication measures [3]. Recently, the high market price of the snakehead’s firm, white, practically boneless flesh with a most agreeable flavor and hardiness to handling has made the culture of this species commercially viable [4].

With the continued growth of aquaculture worldwide, it is essential to search for alternative fish meal sources to guarantee the future development of the aquafeed industry [5,6,7,8,9]. Aquafeeds, in general, have considerably relied on fish meal (FM), which is an optimal protein source to ensure fast growth and good health of farmed fish, especially in carnivorous species [10,11,12,13]. Nevertheless, the reduction in fish landing and continuously increasing demand from the aquaculture sector has made fish meal prices rise over the years [14,15]. The rising cost, added to the uncertain availability of FM supplies, forced nutritionists and feed manufacturers to find inexpensive, abundant, and readily available alternative protein sources as a substitution [16,17,18]. Therefore, the replacement of FM with environmentally sustainable alternative protein sources has been one of the main targets of aquaculture in recent decades [19,20,21].

Recently, insects have been recognized as one of the most potent alternative protein sources for animal feeds, mainly due to their high nutritional composition, easy production, rapid growth, short reproductive cycle, high feed conversion efficiency, and low production cost [18,22,23,24,25,26]. Several studies on the potential usage of insects, such as black soldier fly larvae, house fly maggots, mealworms, grasshoppers, crickets, and silkworm meal, as FM replacements have been reported [24,27]. Among a wide variety of insects, the black cricket (*Gryllus bimaculatus*), also commonly known as the two-spotted cricket, is one of the most abundant cricket species which can be found in the tropical and subtropical regions of Asia, Africa, and Europe [28]. High protein quality, quantity, great feed conversion efficiency, prolific breeding habits, short life cycle, and rapid growth are among many reasons why crickets have long been recognized as promising candidates to constitute inexpensive and sustainable sources of protein for aquaculture [20].

Due to its high nutritional quality, cricket meal has the potential to partially or completely replace fish meal in fish feed [16,17,29]. Cricket meal (CM) has high crude protein and fat content, with up to approximately 64.9 and 17.4%, respectively [20]. Additionally, CM has a significant amount of essential amino acids such as arginine (2.20–3.68%), histidine (0.66–1.94%), lysine (1.71–4.79%), leucine (1.85–5.52%), isoleucine (0.49–3.09%), methionine (0.44–1.93%), cystine (1.0%), phenylalanine (1.0–1.1%), threonine (1.03%), and valine (1.44%) [20]. According to studies, cricket meal and fish meal have nutritional specifications [16,19,24]. However, the proximal nutritional value of insects varies depending on species, developmental stage, or processing methods [28].

There have been many contemporary studies evaluating the use of insects as feed for a variety of various fish species, for example, the common carp, *Cyprinus carpio* [22,30], European seabass, *Dicentrarchus labrax* [31], African catfish, *Clarias gariepinus* [32,33], Nile tilapia, *Oreochromis niloticus* [34,35,36], Atlantic salmon, *Salmo salar* [37,38,39], olive flounder, *Paralichthys olivaceus* [17], Asian Seabass, *Lates calcarifer* [39], and rainbow trout, *Oncorhynchus mykiss* [40]. Nearly all studies to date have focused on the black soldier fly (BSF) with promising results. However, no studies have yet confirmed the efficacy of replacing FM with crickets in formulated diets for the striped snakehead, *Channa striata*. Therefore, this study was designed to evaluate the potential impact of replacing FM protein with CM protein on the digestibility of major nutrients (protein, essential amino acids), growth, feed utilization efficiency, nitrogen and phosphorus retention, and output in striped snakehead juveniles.

## 2. Materials and Methods

### 2.1. Cricket Meal (CM) Preparation

The two-spotted cricket *Gryllus bimaculatus* eggs were incubated for seven days (30 ± 2 °C) in bags. After hatching, the crickets were transferred to plastic boxes (245 cm in length × 125 cm in width × 65 cm in height) in an insect production unit. Water and feed were provided in separate trays. The insect feed was re-processed from Asian Seabass feed (remix waste: 40% protein and 8% fat) processing from an aquafeed factory, pilot plant of the Faculty of Agricultural Technology, King Mongkut’s Institute of Technology Ladkrabang (KMITL), Bangkok Thailand. After 45 days, the crickets, in the imago stage, were harvested using scoop nets and then cleaned using cold water and boiled at 100 °C for 15 min to get rid of contamination. The harvested crickets were kept at minus 20 °C for two weeks until use.

### 2.2. Fish Experiment and Rearing Facilities

Snakehead juveniles with no clinical signs of infectious diseases were purchased from a local fish farm in Singburi province and acclimated for 12 days to laboratory conditions. A total of 225 snakehead (*Channa striata*) juveniles, with an average initial body weight of 15.00 ± 0.01 g fish^−1^ (*n* = 30), were stocked into 9 rectangular aquarium tanks (61 × 76 × 97 cm) within a semi-recirculating system at a stocking density of 25 fish aquaria^−1^ and assigned into 3 treatments, in triplicates of each treatment. During the feeding trial, using the standard protocol of APHA [41], water quality parameters of temperature (28 ± 1 °C), dissolved oxygen (5.66 ± 1.19 mg L^−1^), pH (7.88 ± 0.35), total alkalinity (148.50 ± 38.65 mg L^−1^), water flow rate (6 L h^−1^) were periodically measured at the Department of Fisheries, Faculty of Agriculture, Khonkaen University, Khonkaen, Thailand. For 10 weeks, every day, fish were fed 3% of their body wet weight, twice a day (08:30 a.m. and 4:30 p.m.). After feeding, tanks were cleaned to ensure that water quality remained within acceptable ranges. Feces were collected by siphoning on the next day, while the dead fish were collected and dealt with before starting the daily feeding regime.

### 2.3. Experimental Diets

Thai local tuna FM (grade 60 protein content) was replaced by CM at three levels (CM_0%_, CM_50%_, and CM_100%_). Three experimental diets were investigated in this study; CM_0%_: the control basal diet that used FM at 100% (without CM), while the remaining diets (CM_50%_ and CM_100%_) were the diets that substituted FM by CM at levels of 50% and 100%, respectively. The diet’s ingredients are presented in Table 1.

The dried coarse material was ground using a laboratory mill grinder to generate a fine mill powder to pass through a 300-micron mesh tray on a small shaker sieve system, as described by Chainark et al. [26]. Moreover, by using the standard official methods of analysis as described by AOAC [42], the biochemical composition, whole body composition calcium, phosphorus content, and amino acid profile of FM, CM, and experimental diets (CM_0%_, CM_50%_, and CM_100%_) are presented in Table 2.

All sold ingredients were mixed with a standard laboratory mixer. Liquid ingredients were then added to the blended feed. The moist dough was pelleted by an industrial small-scale extruder machine to produce 2 mm size pellets. The resulting moist pellets were dried at 38 °C for 2 days. The diets were stored in plastic bags in a refrigerator (−4 °C) until use. The diets were formulated to meet the standard of the commercial diet of typical carnivorous and marine fish set by the Department of Fisheries, Thailand (moisture ≤ 10, protein ≥ 42, and fat ≥ 10 g 100 g^−1^).

### 2.4. Growth Performance, Feed Efficiency, and Biometric Indices

Fish were counted at the beginning and the end of the trial to calculate the survival rate (%). The feeding trial ended after 10 weeks, after 24 h of fasting [17]. From each replicate, 5 fish were randomly weighed at the end of the trial to calculate growth performance, feed utilization, and biometric indices. Final body weight (FBW, g), weight gain (WG, g), specific growth rate (SGR, %/day), feed conversion ratio (FCR, feed: gain), hepatosomatic index (HSI, %), and viscerosomatic index (VSI, %) were calculated using the following formulas as labeled beforehand by Prachom et al. [43]:

Weight Gain (WG, g fish^−1^) = FW − IW (1)

(2) $$\mathrm{Specific}\;\mathrm{growth}\;\mathrm{rate}\;(\mathrm{SGR},\;\%/{\mathrm{day}}^{-1})=100\times \left(\frac{\mathrm{Ln}\;\mathrm{FBW}\;-\;\mathrm{Ln}\;\mathrm{IBW}\;}{t}\right)$$

where Ln and t are t natural logarithmic and time in days, respectively.

(3) $$\mathrm{Feed}\;\mathrm{conversion}\;\mathrm{ratio}\;(\mathrm{FCR},\;\mathrm{feed}:\;\mathrm{gain})=\frac{\mathrm{Feed}\;\mathrm{intake}\;\left(g\right)\;}{\mathrm{Body}\;\mathrm{weight}\;\mathrm{gain}\;\left(g\right)\;}$$

(4) $$\mathrm{Survival}\;\mathrm{Rate}\;(\%)=100\times \frac{\;\mathrm{The}\;\mathrm{final}\;\mathrm{number}\;\mathrm{of}\;\mathrm{fish}\;}{\;\mathrm{The}\;\mathrm{initial}\;\mathrm{number}\;\mathrm{of}\;\mathrm{fish}\;}$$

Hepatosomatic index (HSI, %) = liver weight/body weight × 100(5)

Viscerosomatic index (VSI, %) = visceral weight/body weight × 100(6)

### 2.5. Biochemical Composition Analysis

To determine the proximal whole-body analysis at the end of the experiment, 5 fish were randomly selected from each replicate and pulverized, blended, and stored at −20 °C for further examination. Dry matter, crude protein, crude fat, crude ash, phosphorus, and calcium content were all measured according to AOAC [42] guidelines.

### 2.6. Digestibility Trial

The apparent digestibility coefficients (ADCs) of different experimental diets were measured using chromic oxide (Cr_2_O_3_) as an external marker at a level of 5 g kg^−1^ diet. Briefly, after one month of feeding, the fecal samples were collected by hand siphoning from each aquarium every morning before the start of feeding. The feces were collected on filter paper for drying according to the protocol of Prachom et al. [43]. The collected feces were dried in an oven at 50 °C for about 24 h and stored at −20 °C [44] until further chemical analysis. Duplicate groups of 60 fish were fed one of the experimental diets containing 1% chromic oxide (Cr_2_O_3_, Sigma) as an indigestible marker. The digestibility test was run in three periods of 10 days, as previously described in detail [45]. All fecal samples from each tank were pooled in each period and frozen at −80 °C until analyzed for chromic oxide [42,46]. Nitrogen (N) and phosphorus (P) waste output were calculated as described by Lazzari and Baldisserotto [47]. The apparent digestibility coefficients (ADCs) of the experimental diets and ingredients were calculated according to Maynard et al. [48] as described accordingly:

ADC of nutrient, % = 100 − (100 × (dietary Cr_2_O_3_ level/faeces Cr_2_O_3_ level) × (faeces nutrient/dietary nutrient) (7)

ADC of ingredient = ADC test diet + [(ADC test diet − ADC reference diet) × (0.7 × dietary nutrient of reference/0.3 × nutrient of test ingredient)](8)

### 2.7. Amino Acids and Phosphorus Analysis

Amino acids in feed ingredients, diets, and fecal material were determined according to the protocol described by AOAC [42]. As such, dry ground samples were digested with 4 mol L^−1^ methane sulphonic acid (Sigma-Aldrich, St. Louis, MO, USA), and AAs were determined by using a programmed automatic amino acid analyzer (L-8900; Hitachi, Japan). Detection used a post-column derivatization method with ninhydrin (520 nm, for the total acid content). A suite of amino acid concentrations as external standards, including L Norleucine (synthetic amino acid) addition as the internal standard within the sampling protocol and digestion stage, dilution, and injection (20 µL) was employed. However, the apparent nutrient digestibility coefficients (ADCs, %) and Amino Acid profile (% of digestible amino acid basis) of the FM and CM were presented in Table 3.

Apparent net protein utilization (ANPU, %) and phosphorous retention (PR, %) in feeds and fish were determined according to the method of Palma et al. [49]:

Apparent net protein utilization (ANPU, %) = (protein gain/protein intake) × 100(9)

Phosphorus retention (PR, %) = (P gain/P intake) × 100(10)

### 2.8. Statistical Analysis

Statistical analysis was performed using ANOVA analysis. Differences among means were considered significant at *p* < 0.05. Multiple ranges of post hoc comparisons were performed using the least significant difference (LSD) to resolve the differences among the means of replication, according to Duncan, using the SPSS program. GraphPad Prism version 9 was applied to perform the graphical statistical analysis.

## 3. Results

### 3.1. Growth Performance, Feed Utilization, and Some Biometric Indices

Snakeheads receiving diets containing different levels of CM (CM_50%_ and CM_100%_) showed enhanced growth compared to the control diet (Table 4). The superior values of FBW were recorded for fish fed CM_100%_ compared to the control diet (CM_0%_), which showed the lowest values of performance parameters. Furthermore, the FCR values significantly improved with the incremental CM levels (CM_50%_ and CM_100%_) compared to the basal diet (CM_0%_). However, FI, SGR, VSI, HSI, and SR were not significantly affected by CM levels (CM_50%_ and CM_100%_) compared to the basal diet (CM_0%_), as presented in Table 4.

### 3.2. Whole-Body Chemical Composition

As presented in Table 5, the replacement of FM by CM affected the whole-body composition. The findings show a significant difference (*p* < 0.05) in protein, lipid, and ash with diets containing different levels of CM levels (CM_50%_ and CM_100%_) compared to the control diet (CM_0%_). The highest level of protein, lipid, and ash was observed in the group that received 100% CM level (CM_100%_). No significant difference (*p* < 0.05) in phosphorus and calcium content, as presented in Table 5.

### 3.3. Apparent Digestibility Coefficient (ADC) and Amino Acids Profile of the Experimental Diets

Apparent digestibility coefficient (ADCs) values of dry matter, protein, fat, and phosphorus increased significantly by CM inclusion in the diet in comparison with the control diet. The highest ADC_prot_ (91.95%) was found at the CM_100%_. Similarly, the highest ADC_fat_ and ADC_P_ (90.80% and 95.42%, respectively) were recorded at CM_100%_. However, ADC_Calcium_ calcium digestibility decreased significantly with CM inclusion level compared to the control diet (Table 6). On the other hand, apparent digestibility coefficients of digestible amino acids (EAAs and NEAAs) were significantly (*p* < 0.05) increased with increasing CM levels (CM_50%_ and CM_100%_) compared to the control diet (CM_0%_), as presented in Table 6.

### 3.4. Apparent Net Protein Utilization and Phosphorus Retention

**Figure 1:**
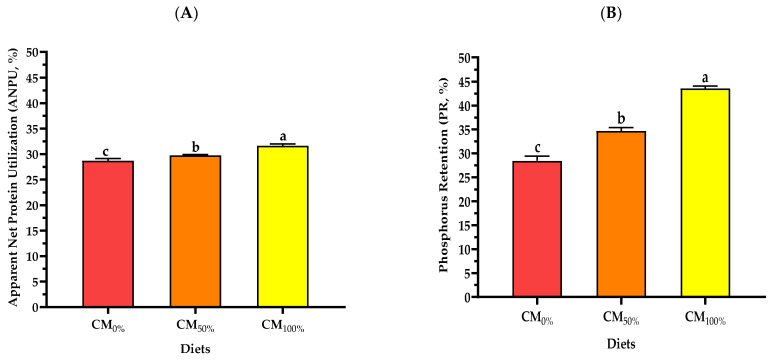
Apparent net protein utilization (**A**) and phosphorus retention (**B**) of snakehead feed diets containing different levels of cricket meal for *Channa striata*. CM_0%_: the control basal diet that used FM at 100% (without CM); CM_50%_ and CM_100%_: the diets that substituted FM with CM at levels of 50% and 100%, respectively. Data were represented as means ± SD (*n* = 5). Different letters in each row indicate significant differences (*p* < 0.05).

Fish fed on CM_100%_ recorded superior significant values (*p* < 0.05) of apparent net protein utilization (ANPU) and phosphorus retention (PR) compared to the control diets (CM_0%_) (Figure 1). The highest ANPU and PR level was found in fish fed with the increasing inclusion level of CM.

### 3.5. Nitrogen and Phosphorus Waste Output Estimation (g kg^−1^ of Fish Biomass)

Total waste, solid waste, dissolved waste, and digestibility of nitrogen and phosphorus were presented in Table 7. For nitrogen (N), The amount of N load (g kg^−1^ of fish biomass) was 71.80, 69.02, and 65.41 (g kg^−1^ of fish biomass^−1^) for CM_0%_, CM_50%_, and CM_100%_, respectively. This indicates a decrease in the amount of nitrogen excreted with the increase in CM levels. Additionally, this is found with a high digestibility of nitrogen. Similarly, in the case of phosphorus (P), the amount of P load (g kg^−1^ of fish biomass^−1^) was 16.82, 11.92, and 8.25 for CM_0%_, CM_50%_, and CM_100%_, respectively, and also exhibited decreases in P digestibility with the increase in CM levels (Table 7).

## 4. Discussion

### 4.1. Growth Performance, Feed Utilization, and Some Biometric Indices

The current investigations showed that growth performance and protein utilization increased when the FM component was replaced from 50 to 100% by CM. Results indicated that the 100% substitution of FM with CM had no adverse effect on fish growth and feed conversion under controlled experimental conditions. The present findings are consistent with those of Magalhães et al. [50], Iaconisi et al. [51], and Mastoraki et al. [23], where various insect larval meals were evaluated in experimental diets to replace FM for European seabass, *Dicentrarchus labrax*, and blackspot seabream, *Pagellus bogaraveo*. Similarly, the two-spotted cricket can substitute up to 100% of FM protein in the diet of African catfish, *Clarias gariepinus*, and whiteleg shrimp, *Litopenaeus vannamei*, juveniles without deleterious effects on growth performance, amino acid profile, and nutrient digestibility [24]. In addition, Taufek et al. [32] found that African catfish fed diets containing CM significantly improved growth and feed efficiency. In contrast with these findings, Sanchez-Muros et al. [52], Taufek et al. [33], and Jeong et al. [20] found that olive flounder juveniles and other species showed a negative association with the replacement of FM protein by insect meal, up to 20% inclusion; growth performance tended to decline after that threshold. This discrepancy could be attributed to different factors such as (i) the differences in nutrient requirements among species, and (ii) differences in physiology and anatomical systems of different fish species that could affect their capacity to utilize dietary nutrients, particularly carbohydrates, due to their mode of nutritional assimilation. Nevertheless, the extent to which FM can be replaced with insect meal without impacting growth performance and feed utilization is very much dependent on several factors such as fish species, insect species, life stage, and diet composition [20,53]. The current findings could be attributed to different reasons: (i) excellent profile of AAs in spotted cricket meal; (ii) high nutritive value; (iii) high protein quality; (iv) better feed utilization; (v) spotted cricket contains functional chitinous materials that could have positive impacts on modulating gastrointestinal microbiota, and consequently promote performance and feed utilization efficiency [54]; (vi) the improvement in nutrient digestibility values of fish fed diets containing CM; (vii) the increase in CM and nutrient digestibility values of fish fed diets supplemented with CM improves the FCR level. No significant differences were observed in the visceral somatic index (VSI) and the hepatic somatic index (HSI) among all fish groups. Similarly, Zhou et al. [55], Mastoraki et al. [23], and Hoffmann et al. [56] found no significant differences in the VSI and HSI in European sea bass, *D. labrax* fed with the diets with inclusion levels of black soldier fly larvae meal. In addition, Wang et al. [57] reported that for Japanese seabass, *Lateolabrax japonicus,* no significant changes in somatic condition for seabass larvae fed black soldier fly were apparent. The current study showed that the survival rate was significantly increased when the FM component was replaced to 100% by CM. However, our results disagree with the results obtained by Hanan et al. [16], who did not find significant improvement in the survival rate of hybrid red tilapia (*Oreochromis* spp.) when replacing FM with CM (*Gryllus bimaculatus*) at a level of 100%. In addition, the same results of Hanan et al. [16] were previously reported by Jeong et al. [20], who found that no significant improvement in the survival rate of the juveniles of olive flounder, *Paralichthys olivaceus*, when replacing FM with CM (*Gryllus bimaculatus*) at a level of 80%. This opposition may be attributed to different factors such as different species, experimental conditions, and the discrepancy in nutrient requirements among species.

### 4.2. Whole-Body Chemical Composition

The body composition of fish may be affected by many factors, including age, size, the ingredient used in the diets [58], and water temperature [59]. Apart from that, the feeding rate could also affect the body composition of fish [33]. The results in this current study showed that the gross body composition of snakeheads fed with 50–100% CM inclusion level showed significantly higher crude protein content and fat content compared to the control. The present findings are consistent with those reported by Taufek et al. [33], which stated that full replacement of CM up to 100% of FM affected the whole-body composition of African catfish. Belforti et al. [60] and Kroeckel et al. [61] showed that whole-body moisture and fat content tended to decline while the protein content tended to increase with high cumulative levels of black soldier meal diets for rainbow trout and turbot juveniles. In contrast with the present study, Jeong et al. [20] and Guerreiro et al. [28] reported that no changes were evident in the whole body and fillet proximate composition (*p* ≥ 0.05) when the CM level increased for olive flounder, *Paralichthys olivaceus*, juvenile diets. In a similar study, Zhou et al. [55] found no variations of lipid and protein body content in the whole body of common carp and European sea bass receiving different black soldier fly levels. Furthermore, Gasco et al. [62] reported no differences between the carcass compositions of European sea bass fed FM and different levels of the meal of the yellow mealworm beetle, *Tenebrio molitor* (TM). Fluctuations of CP and fat contents could be attributed to (i) fish species differences and (ii) the changes in enzyme activities involved in some metabolic processes such as protein synthesis and degradation in muscle and liver [27].

### 4.3. ANPU, ADC, and Amino Acids Digestibility

In the present study, the CM was shown to be a good supplier of digestible histidine, threonine, leucine, valine, phenylalanine, and arginine, among several essential amino acids based on their digestible amino acid data. The digestible amino acid values (Table 3) show that higher digestibility of amino acids in CM appears to improve their availability compared to their total levels in this component, as observed for both isoleucine and leucine. It should be noted that levels of most EAAs are relatively higher in CM than in FM (tuna fish meal). Digestibility values for arginine and histidine ranged from 2.71 to 3.98% and 0.84 to 1.36%, respectively, being higher at 100% FM substitution (24% dietary CM inclusion). There is also an elevation of digestible lysine from 2.96–3.55% at the higher CM level. In contrast, the results showed a progressive reduction in the digestible methionine level of diets with increasing CM substitution from 0.97% in the FM control to 0.84% for maximum CM incorporation. Furthermore, the essential amino acid (EAA) levels reported in this study were comparable with the results reported by Wang et al. [57] on *G. testaceus*. However, in the latter study, the authors reported a higher level of lysine and methionine (8.35% and 1.98%, respectively) with a diet of 100% CM compared to Jayanegara et al. [63] and Oibiokpa et al. [64] but on a gross amino acid basis. These researchers reported levels of lysine (6.59% and 5.29%, respectively) and methionine (1.88% and 2.29%, respectively) in CM-based diets. Different levels of essential amino acids (EAA) may vary depending on the insect’s diet and life stages [16,27]. This should be a prime consideration in strategic work to evaluate insect meal for fish, but it is not always considered in feeding trial investigations. For these reasons, it would be useful to report essential amino acids as a percent of protein (g 100 N × 6.25 g^−1^) in future assessments for direct comparisons with other protein sources, such as fish meal and soybean meal, due to potential variations of EAA composition with different substrates used in CM production. Additionally, it should caution that the chitin component of the exoskeletal insect carapace is a confounding issue as the N content can mislead the true protein content if a nitrogen conversion factor of ×6.25 is employed for assessing the crude protein content of cricket meal. This consideration can lead to significant discrepancies in formulating iso-nitrogenous-based diets for all insect-related protein biomass and affect amino acid comparisons. Insects, like other animals, are high in protein, lipids, vitamins, and minerals, though the levels of these nutrients may vary depending on the animal’s diet and life stages (larva, pupa, prepupa, and imago) [27]. Different nutrient levels in FM might occur since there are two varieties of it: those made from fishery waste linked with the processing of various fishery products (salmon, tuna, etc.) and those made from fish harvested specifically to make FM (herring, menhaden, pollock, etc.) [16]. Several trials have explored insect meal as a potential candidate dietary ingredient for different fish species [27,36,62], but locusts and crickets have received much less attention than the black soldier fly (*Hermetia illucens*). The current trial signifies one of the first attempts to evaluate the viability of using CM to replace FM either partially or completely in dietary formulations for snakehead (*C. striata*) juveniles. Essential and non-essential amino acid profiles of FM and CM were comparable to estimated requirements for snakehead in terms of their balance (Table 5).

In the present study, the lipid content in the CM obtained in this analysis was found to be 19.9% and typical of levels found in other investigations. Similar results were also reported by Miech et al. [65], Perera and Bhujel [36], and Jeong et al. [20]. On the other hand, the protein content is like that of *G. bimaculatus*, at 59.9% [16]. Cricket meal is seen to have a high nutritional value of their components, with a high proportion of protein. However, crude protein levels in CM and FM were lower (60.7 and 56%, respectively) than those described by Jeong et al. [20] (64.9 and 72.3%, respectively) but comparable with Perera and Bhujel’s [36], which were 56.6 and 56.0%; respectively. Different types of food consumed by crickets are a factor contributing to and reflecting differences in nutrient values [66]. It was an interesting observation that the non-essential amino acid profile was also elevated in diets with CM, and this amino acid (such as hydroxyproline) is found in connective tissue proteins such as collagen and elastin [67] in fish as well as major vertebrate species. Overall, coupled with the superior digestibility coefficients of the amino acids in CM, it is apparent that it offers an excellent balanced amino acid availability for snakeheads. Many other studies reported failing to provide such a detailed appraisal of fish fed on insect meals [32,50,51].

In the current investigation, it was evident that CM was more digestible than FM for snakeheads in terms of crude protein, amino acids, crude fat, dry matter, and phosphorus (Table 5). The results of the ADC of protein of CM (90.4%) and FM (89.3%) agreed with Fagbenro [68], who found that the ADC of different plant and animal proteins ranged between 58 and 92%. Moreover, Hossain et al. [69] found that rohu, *Labeo rohita*, digest the protein of non-defatted and defatted silkworm pupae higher than the protein of FM. Additionally, Hossain et al. [70] found that Mozambique tilapia, *Oreochromis mossambicus*, can digest the protein of silkworm pupae with an efficiency of 86%. Contrary to the present study, previous research conducted on Nile tilapia and African catfish by Alegbeleye et al. [71], Jabir et al. [72], and Bosch et al. [73] using superworm meal and grasshopper meal showed significantly lower overall nutrient digestibility, probably due to varying fiber content as principal chitin in the cutin component of the exoskeleton. The present study with snakehead showed that fish fed on diets containing FM had a lower ADC of dry matter, crude protein, and phosphorus compared to those fed CM. This observation conformed to the previous studies. Additionally, the high ADC content of crude fat in the CM diets indicates the capacity of snakeheads to assimilate the fat component of CM as an energy source [71,74].

### 4.4. Nitrogen and Phosphorus Waste Output Estimation (g kg^−1^ of Fish Biomass)

It should be cautioned that digestibility coefficients derived for the diets were based on the classical fixed ratio of 70:30 (reference diet: test ingredient ratio). Although a standard approach commonly used by many researchers, this may not provide accurate estimates for specific nutrients due to varying ingredient level ratios. Additionally, the collecting of feces may overestimate digestibility coefficients due to nutrient leaching losses [73]. Recently, considerable attention has been given to limiting inputs of nitrogen (N) and phosphorus (P) in fish feed to fish as means of reducing environmental loads. The present investigation with snakehead showed that replacing FM with insect meal reduced the total biological (metabolic) output of phosphorus and nitrogen, which consequently would minimize nitrogen and phosphorus loading into the environment. The present results could be attributed to the following reasons: (i) P content in CM is lower than in FM [30], (ii) P in insects is likely to be readily available [68,75], and (iii) high dietary inclusion of CM improved P and N digestibility and whole-body retention.

## 5. Conclusions

In conclusion, the results indicated that protein from cricket meal was well digested and assimilated by snakehead (*Channa striata*) juveniles at a marginally superior level than fish meal. CM was therefore deemed to be an acceptable alternative feed ingredient suitable for snakehead feed formulations supporting growth performance. The findings suggest that the substitution of FM by CM at a dietary inclusion level of up to 100% (equating to 45% dietary inclusion) is achievable with many benefits. More studies are warranted to test other types of locally abundant insects which might serve as important ingredients to replace FM, which is expensive and being used more strategically in formulated practical diets. Currently, the commercial CM production cost is much higher than FM on a per unit of protein basis. Inevitably, future scale-up of production volume will become technically and financially feasible, thus reducing the price of CM. Cricket meal offers a potential solution to the ‘protein gap’ as it meets the needs of the circular economy and would be a viable sustainable commodity for application in modern aquafeeds for many other farmed species when costs can be reduced.

## Figures and Tables

**Table 1:** Ingredient, proximate composition, and replacement levels of the experimental diets on striped snakehead (*Channa striata*) juveniles.

Diet Ingredients	Experimental Diets		
	CM_0%_	CM_50%_	CM_100%_
Tuna fish meal, grade 60 protein	45.0	22.5	0.0
Cricket meal (*G. bimaculatus*)	0.0	22.5	45.0
Dehulled soybean meal	24.00	24.00	24.00
Wheat feed flour	23.00	23.00	23.00
Tuna fish solubles	2.00	2.00	2.00
Tuna fish oil	2.00	2.00	2.00
Vitamins and minerals premix *	0.5	0.5	0.5
Mono-calcium phosphate	2.0	2.0	2.0
Chromic oxide (Cr_2_O_3_)	0.50	0.50	0.50
Antioxidants *	0.50	0.50	0.50
Antimicrobial agents *	0.50	0.50	0.50

CM_0%_: the control basal diet that used FM at 100% (without CM); CM_50%_ and CM_100%_: the diets that substituted FM with CM at levels of 50% and 100%, respectively. * Vitamin–mineral premix (U kg^−1^): A: 12,000,000 IU, D3: 2,200,000 IU, E: 100,000 mg, K_3_: 12,000 mg, B_1_: 25,000 mg, B_2_: 25,000 mg, B_6_: 23,000 mg, B_12_: 43 mg, Pantothenic: 75,000 mg, Niacin: 125,000 mg, Folic acd: 4000 mg, Biotin: 800 mg, Copper: 80 mg, Iron: 150 mg, Manganese: 50 mg, Zinc: 120 mg, and Selenium: 0.3 mg. Antioxidant (butylated hydroxyl toluene and butylated hydroxyl anisole). Antimicrobial agents (formic acid, propionic acid, and benzoic acid).

**Table 2:** Proximate compositions and amino acid profile (%) of ingredients and practical diets used in this study on striped snakehead (*Channa striata*) juveniles during the experimental period.

Amino Acids	FM	CM	Experimental Diets		
			CM_0%_	CM_50%_	CM_100%_
Proximate compositions (%)					
Dry matter	89.2 ± 0.12	92.90 ± 0.35	92.5 ± 0.07	92.5 ± 0.12	92.8 ± 0.12
Crude protein	56.0 ± 0.10	60.70 ± 0.25	42.50 ± 0.15	42.6 ± 0.28	42.4 ± 0.21
Crude fat	10.0 ± 0.15	19.90 ± 0.10	10.60 ± 0.07	12.40 ± 0.10	13.5 ± 0.15
Calcium	3.02 ± 0.12	0.30 ± 0.15	2.90 ± 0.10	1.70 ± 0.20	1.19 ± 0. 31
Phosphorus	0.88 ± 0.14	0.80 ± 0.10	1.80 ± 0.11	1.50 ± 0.18	1.29 ± 0.15
Amino Acids profile (g 100 g^−1^)					
EAA					
Arginine	3.47	4.59	2.71	3.34	3.98
Histidine	2.18	3.59	0.84	1.10	1.36
Isoleucine	2.60	1.88	1.76	1.86	1.97
Leucine	4.23	3.96	3.09	3.34	3.59
Lysine	4.49	3.02	2.96	3.25	3.55
Methionine	1.42	0.98	0.97	0.90	0.84
Phenylalanine	2.75	4.52	1.89	1.91	1.92
Threonine	2.63	4.34	1.67	1.69	1.71
Valine	2.88	2.85	2.28	2.33	2.37
Sum of EAA	26.65	29.73	18.17	19.72	21.29
NEAA					
Alanine	5.88	3.26	2.16	2.71	3.26
Aspartic acid	5.22	4.14	3.65	4.36	5.07
Cysteine	0.60	1.00	0.60	0.54	0.47
Glutamic acid	7.38	5.08	5.86	5.46	5.06
Glycine	4.05	2.68	2.47	2.45	2.43
Proline	3.64	4.18	2.81	3.45	4.09
Serine	3.48	5.74	2.37	2.32	2.26
Tyrosine	1.86	3.48	1.28	1.69	2.10
Sum of NEAA	32.11	29.56	21.20	22.98	24.74
Sum of Total AA	58.76	59.92	41.37	42.70	46.03

FM: fish meal, CM: cricket meal (*G. bimaculatus*), CM_0%_: the control basal diet that used FM at 100% (without CM); CM_50%_ and CM_100%_: the diets that substituted FM with CM at levels of 50% and 100%, respectively; EAA: essential amino acids; NEAA: non-essential amino acids. Data of the proximate compositions were represented as means (*n* = 3).

**Table 3:** Apparent digestibility coefficients (ADCs, %) of the test ingredient two-spotted cricket (*Gryllus bimaculatus*) meal and fish meal.

Apparent Nutrient Digestibility Coefficient (%)	FM	CM
Dry matter	73.1 ± 0.1	77.6 ± 0.4
Crude protein	89.3 ± 0.1	90.4 ± 0.5
Crude fat	82.8 ± 0.5	89.8 ± 0.1
Calcium	86.6 ± 0.0	86.1 ± 0.1
Phosphorus	84.2 ± 0.5	95.7 ± 0.1
Amino Acid Profile (% of digestible amino acid basis)		
EAA		
Arginine	87.9 ± 0.1	92.3 ± 0.5
Histidine	84.5 ± 0.1	91.8 ± 0.1
Isoleucine	83.5 ± 0.6	87.0 ± 0.2
Leucine	85.5 ± 0.2	88.8 ± 0.4
Lysine	91.0 ± 0.5	97.4 ± 0.6
Methionine	84.4 ± 0.4	95.6 ± 0.0
Phenylalanine	87.1 ± 0.3	95.9 ± 0.0
Threonine	83.6 ± 0.2	86.9 ± 0.1
Valine	77.4 ± 0.2	77.7 ± 2.0
NEAA		
Alanine	90.5 ± 0.2	100.2 ± 0.1
Aspartic acid	82.9 ± 0.4	88.5 ± 0.4
Cysteine	80.5 ± 0.2	95.3 ± 0.1
Glutamic acid	88.7 ± 0.7	99.4 ± 0.2
Glycine	84.7 ± 0.2	93.3 ± 0.8
Proline	82.8 ± 0.1	85.2 ± 0.1
Serine	83.9 ± 0.1	84.5 ± 0.4
Tyrosine	76.5 ± 0.2	81.7 ± 0.2

FM: fish meal (tuna fish meal); CM: cricket meal (*G. bimaculatus*); EAA: essential amino acids; NEAA: non-essential amino acids. Data were represented as means ± SD (*n* = 3).

**Table 4:** Growth performance, feed utilization efficiency, and body indices values of striped snakehead (*Channa striata*) fed with experimental diets during the experimental period.

Parameters	Experimental Diets		
	CM_0%_	CM_50%_	CM_100%_
FBW (g fish^−1^)	55.50 ± 1.00 ^b^	57.00 ± 0.50 ^a^	57.80 ± 0.30 ^a^
WG (g fish^−1^)	40.00 ± 0.50 ^c^	42.00 ± 0.50 ^b^	43.40 ± 0.045 ^a^
FCR (feed: gain)	1.45 ± 0.02 ^a^	1.39 ± 0.01 ^ab^	1.35 ± 0.03 ^c^
SGR (% day^−1^)	1.87 ± 0.04	1.91 ± 0.22	1.93 ± 0.33
VSI (%)	5.21 ± 0.45	5.20 ± 0.29	5.19 ± 0.32
HSI (%)	0.90 ± 0.07	0.87 ± 0.03	0.86 ± 0.28
SR (%)	84.40 ± 0.60 ^b^	84.60 ± 1.00 ^b^	86.70 ± 1.00 ^a^

CM_0%_: the control basal diet that used FM at 100% (without CM); CM_50%_ and CM_100%_: the diets that substituted FM with CM at levels of 50% and 100%, respectively; FBW: final body weight (g fish^−1^); WG: weight gain (g fish^−1^); FCR: feed conversion ratio (feed: gain); SGR: specific growth rate (% day^−1^); SR: survival rate (%), HSI: hepatosomatic index (%); and VSI: viscerosomatic index (%). Data were represented as means ± SD (*n* = 5). Different letters in each row indicate significant differences (*p* < 0.05). The absence of letters indicates no significant differences.

**Table 5:** Whole body composition (% wet weight basis) of striped snakeheads (*Channa striata*) fed with experimental diets during the experimental period.

Parameters	Experimental Diets		
	CM_0%_	CM_50%_	CM_100%_
Moisture	72.5 ± 1.0	72.2 ± 1.1	72.2 ± 1.0
Crude protein	15.9 ± 0.3 ^c^	16.7 ± 0.3 ^b^	17.4 ± 0.3 ^a^
Crude lipid	7.6 ± 0.0 ^c^	8.8 ± 0.1 ^b^	9.2 ± 0.1 ^a^
Crude ash	1.5 ± 0.0 ^c^	2.6 ± 0.0 ^b^	2.9 ± 0.0 ^a^
Phosphorus	0.9 ± 0.0	0.9 ± 0.0	0.9 ± 0.0
Calcium	1.1 ± 0.0	1.1 ± 0.0	1.1 ± 0.0

CM_0%_: the control basal diet that used FM at 100% (without CM); CM_50%_ and CM_100%_: the diets that substituted FM with CM at levels of 50% and 100%, respectively. Data were represented as means ± SD (*n* = 5). Different letters in each row indicate significant differences (*p* < 0.05). The absence of letters indicates no significant differences.

**Table 6:** Apparent digestibility coefficients (ADCs, %) of striped snakehead (*Channa striata*) fed with experimental diets during the experimental period.

Parameters	Experimental Diets		
	CM_0%_	CM_50%_	CM_100%_
ADCs of Proximate Analysis (%)			
Dry matter	66.0 ± 0.62 ^c^	82.50 ± 0.85 ^b^	83.70 ± 0.50 ^a^
ADC_Protein_	88.0 ± 0.85 ^b^	91.80 ± 0.33 ^a^	91.95 ± 0.50 ^a^
ADC_Fats_	85.30 ± 1.02 ^b^	90.10 ± 0.55 ^a^	90.80 ± 0.58 ^a^
ADC_Phosphorus_	92.40 ± 1.03 ^b^	95.33 ± 0.70 ^a^	95.42 ± 0.14 ^a^
ADC_Calcium_	88.44 ± 0.59	85.90 ± 0.56	86.60 ± 0.50
ADCs of Amino Acid (% of digestible amino acid basis)			
EAA			
Arginine	81.60 ± 0.10 ^b^	89.95 ± 0.18 ^a^	90.51 ± 1.20 ^a^
Histidine	73.40 ± 1.70 ^b^	84.75 ± 0.45 ^a^	85.71 ± 1.07 ^a^
Isoleucine	79.90 ± 1.10 ^b^	85.50 ± 0.10 ^a^	86.55 ± 1.12 ^a^
Leucine	81.62 ± 0.15 ^b^	86.65 ± 0.56 ^a^	87.55 ± 0.85 ^a^
Lysine	86.55 ± 1.50 ^c^	92.27 ± 0.25 ^b^	93.42 ± 0.42 ^a^
Methionine	86.58 ± 0.10 ^b^	87.5 ± 0.36 ^a^	87.6 ± 0.45 ^a^
Phenylalanine	79.15 ± 0.7 ^b^	89.25 ± 0.29 ^a^	89.90 ± 0.75 ^a^
Threonine	79.30 ± 0.35 ^b^	84.70 ± 0.65 ^a^	85.70 ± 0.75 ^a^
Valine	77.69 ± 1.10 ^b^	78.10 ± 0.45 ^b^	79.65 ± 1.02 ^a^
NEAA			
Alanine	69.40 ± 1.00 ^c^	72.30 ± 1.4 ^b^	76.10 ± 0.60 ^a^
Aspartic acid	75.70 ± 0.90 ^b^	83.11 ± 0.45 ^a^	84.20 ± 1.11 ^a^
Cysteine	68.40 ± 0.55 ^a^	86.70 ± 0.36 ^a^	87.61 ± 2.40 ^a^
Glutamic acid	81.04 ± 0.40 ^b^	88.56 ± 0.79 ^a^	90.30 ± 2.7 ^a^
Glycine	72.00 ± 0.50 ^b^	80.75 ± 0.50 ^a^	82.00 ± 2.50 ^a^
Proline	74.70 ± 0.00 ^b^	80.45 ± 0.45 ^a^	81.75 ± 1.20 ^a^
Serine	80.10 ± 1.75	82.90 ± 2.10	84.0 ± 0.90
Tyrosine	64.90 ± 0.57 ^b^	75.30 ± 0.90 ^a^	76.90 ± 0.90 ^a^

ADCs: Apparent digestibility coefficient; EAA: Essential amino acids; NEAA: non-essential amino acids. CM_0%_: the control basal diet that used FM at 100% (without CM); CM_50%_ and CM_100%_ were the diets that substituted FM with CM at levels of 50% and 100%, respectively. Data were represented as means ± SD (*n* = 5). Different letters in each row indicate significant differences (*p* < 0.05). The absence of letters indicates no significant differences.

**Table 7:** Nitrogen waste output estimation (g kg of fish biomass^−1^) of snakehead *Channa striata* fed diets containing different levels of two-spotted cricket meal.

Nutrient Source	Parameters	Experimental Diets		
		CM_0%_	CM_50%_	CM_100%_
Nitrogen	Total waste	71.80 ± 0.22 ^a^	69.02 ± 0.80 ^b^	65.41 ± 0.14 ^c^
	Dissolved waste	60.50 ± 1.52 ^ab^	61.22 ± 1.21 ^a^	58.30 ± 0.21 ^b^
	Solid waste	11.30 ± 0.23 ^a^	7.83 ± 0.21 ^b^	7.11 ± 0.33 ^c^
	Consumed	97.22 ± 0.55 ^a^	94.52 ± 0.94 ^b^	91.82 ± 0.91 ^c^
	Digested	86.83 ± 0.94 ^a^	85.50 ± 0.51 ^ab^	84.5 ± 0.92 ^b^
	Retained	25.40 ± 0.21 ^c^	25.81 ± 0.22 ^b^	26.24 ± 0.22 ^a^
Phosphorus	Total waste	16.82 ± 0.12 ^a^	11.92 ± 0.75 ^b^	8.25 ± 0.13 ^c^
	Dissolved waste	14.84 ± 0.21 ^a^	10.9 ± 0.1 ^b^	7.52 ± 0.30 ^c^
	Solid waste	2.05 ± 0.25 ^a^	1.43 ± 0.30 ^b^	0.72 ± 0.10 ^c^
	Consumed	25. 72 ± 0.35 ^a^	20.9 ± 0.1 ^b^	16.26 ± 0.41 ^c^
	Digested	23.85 ± 0.95 ^a^	19.9 ± 0.9 ^b^	15.57 ± 0.55 ^b^
	Retained	9.40 ± 0.31 ^a^	9.10 ± 0.25 ^a^	8.30 ± 0.21 ^b^

CM_0%_: the control basal diet that used FM at 100% (without CM); CM_50%_ and CM_100%_: the diets that substituted FM with CM at levels of 50% and 100%, respectively. Data were represented as means ± SD (*n* = 5). Different letters in each row indicate significant differences (*p* < 0.05).

## Data Availability

Not applicable.
